# Senescence in Adipose-Derived Stem Cells: Biological Mechanisms and Therapeutic Challenges

**DOI:** 10.3390/ijms25158390

**Published:** 2024-08-01

**Authors:** Riccardo Foti, Gabriele Storti, Marco Palmesano, Maria Giovanna Scioli, Elena Fiorelli, Sonia Terriaca, Giulio Cervelli, Bong Sung Kim, Augusto Orlandi, Valerio Cervelli

**Affiliations:** 1 Plastic Surgery, Department of Surgical Sciences, University of Rome “Tor Vergata”, 00133 Rome, Italy; riccardofoti.md@gmail.com (R.F.); marcopalmesano@gmail.com (M.P.); valeriocervelli@virgilio.it (V.C.); 2 Anatomy Pathology Institute, Department of Biomedicine and Prevention, University of Rome “Tor Vergata”, 00133 Rome, Italy; scioli@med.uniroma2.it (M.G.S.); elena.fiorelli1@gmail.com (E.F.); terriacasonia093@gmail.com (S.T.); orlandi@uniroma2.it (A.O.); 3 Department of Experimental Medicine, University of Rome “Tor Vergata”, 00133 Rome, Italy; giulio.cervelli@gmail.com; 4 Department of Plastic Surgery and Hand Surgery, University Hospital Zurich, 8006 Zurich, Switzerland; bong-sung.kim@usz.ch

**Keywords:** adipose-derived stem cells, mesenchymal stem cells, ageing, senescence, stem cell therapy, diabetes, senolytic drugs

## Abstract

Adipose tissue-derived stem cells (ADSCs) represent a subset of the mesenchymal stem cells in every adipose compartment throughout the body. ADSCs can differentiate into various cell types, including chondrocytes, osteocytes, myocytes, and adipocytes. Moreover, they exhibit a notable potential to differentiate in vitro into cells from other germinal lineages, including endothelial cells and neurons. ADSCs have a wide range of clinical applications, from breast surgery to chronic wounds. Furthermore, they are a promising cell population for future tissue-engineering uses. Accumulating evidence indicates a decreased proliferation and differentiation potential of ADSCs with an increasing age, increasing body mass index, diabetes mellitus, metabolic syndrome, or exposure to radiotherapy. Therefore, the recent literature thoroughly investigates this cell population’s senescence mechanisms and how they can hinder its possible therapeutic applications. This review will discuss the biological mechanisms and the physio-pathological causes behind ADSC senescence and how they can impact cellular functionality. Moreover, we will examine the possible strategies to invert these processes, re-establishing the full regenerative potential of this progenitor population.

## 1. Introduction

Adipose tissue is an excellent source of mesenchymal stem cells (MSCs), owing to its abundance of adipose-derived MSCs (ADSCs) that can be easily accessed with minimal invasiveness [1,2,3]. ADSCs demonstrate impressive durability throughout extended periods of culture, smooth expansion in vitro, and the ability to differentiate into diverse cell lineages, such as adipogenic, osteogenic, chondrogenic, and angiogenic cells [4,5]. ADSCs are characterized by their fibroblast-like morphology, colony-forming properties, self-renewal capacity, and significant proliferative rate [6]. This distinctive profile positions them as an attractive tool for tissue engineering and regenerative medicine applications [7,8].

The harvesting of ADSCs is considered an easy and minimally invasive procedure that can be performed with minimal risk to the patient. It typically involves subcutaneous lipoaspiration through small skin incisions. Adipose tissue ensures a high cell yield and, compared to the harvesting of inter alia bone marrow-derived stem cells, this procedure takes less time and provides less discomfort to the patient [9,10].

Mesenchymal stem cells derived from bone marrow, adipose tissue, and umbilical cord blood exhibit nearly indistinguishable characteristics when assessing their morphology, immune phenotype, isolation success, colony formation, and differentiation capacity [11].

ADSCs are recognized for their multi-differentiation capabilities that are similar to those of BMSCs (bone marrow stem cells) and demonstrate a high stability and proliferation capacity even in late passages [9,12,13].

Many applications involve the clinical use of adipose tissue and ADSCs. They encompass a wide range of clinical conditions, including chronic wounds, breast surgery, aesthetic surgery, burns, osteocartilaginous reconstruction, and autoimmune diseases [2,5,8,14,15,16,17,18]. Many clinical studies have evaluated the use of autologous or allogeneic ADSCs in treating clinical manifestations of chronic conditions such as Crohn’s disease, critical limb ischemia, and diabetic foot ulcers [19,20]. In wound healing, for example, these cells accelerate tissue repair and re-epithelization to varying extents. The biological contribution of ADSCs may occur through their capacity to proliferate and differentiate or via the secretion of various growth factors and cytokines implicated in these phases [21].

Moreover, ADSCs have exhibited diverse potential applications in ophthalmic conditions. Several publications from various research groups have underscored their potential advantages, especially concerning their immunomodulatory properties. They have demonstrated efficacy in ameliorating corneal scars, augmenting corneal transparency, and fostering the formation of new organized collagen within the host stroma [22].

Furthermore, ADSCs have proven efficacy in addressing symptoms of knee osteoarthritis, as evidenced by improvements in both function and pain levels. Moreover, injections of ADSCs have demonstrated enhancements in cartilage integrity, suggesting the potential for regenerating knee cartilage [23].

Huang T et al. conducted a study to analyze the efficacy and mechanism of adipose-derived mesenchymal stem cell exosome (ADSC-exosome)-mediated protection against methotrexate (MTX)-induced neuronal damage. The morphological and functional recovery of rat hippocampal neurons treated with ADSC-exosomes was assessed via Nissl staining and the modified neurological severity score (mNSS). ADSC-exosome treatment restored hippocampal neuronal cell activity, reduced ROS production, and inhibited apoptosis. In vivo, ADSC-exosomes improved MTX-induced hippocampal neuron damage by activating the Nrf2-ARE pathway, decreasing IL-6, IFN-γ, and TNF-α levels, reducing the amount of TUNEL-positive cells in the hippocampus, and repairing neuronal damage [24].

Vialle EN et al. conducted a study to evaluate the effects of human adipose tissue-derived stem cell (ADSC) infusion via the cauda equina in rats with traumatic spinal cord injury (SCI). The study demonstrates that ADSC infusion mitigates the neuronal loss in SCI, indicating therapeutic potential without affecting myelin preservation or astrocyte proliferation [25].

Hu X et al. demonstrated that ADSC-EVs reduce brain atrophy and improve neuromotor and cognitive functions after ischemic stroke in mice. They found that ADSC-EV injections reduce the loss of oligodendrocytes, increase angiogenesis, promote the M2 repair-associated phenotype, and decrease the levels of the M1 pro-inflammatory phenotype in microglia. In summary, ADSC-EVs reduce ischemic brain injury by regulating microglial polarization, with miRNAs in ADSC-EVs targeting PTEN and STAT1 [26].

Many of the described conditions are chronic and often determine an inflammatory state, which determines accelerated senescence [27]. Moreover, many treated patients are elderly adults presenting these conditions later in their clinical history. Despite these cells having demonstrated a significant variety of therapeutic applications, many studies have shown a decrease in their function that is related to many causes. It is becoming increasingly evident that their therapeutic potential may face limitations due to senescence, the age of the donor, diabetes, metabolic syndrome, and other factors [28].

Senescence refers to the state of irreversible growth arrest that cells undergo as they age or due to specific stressors. It is a cellular response characterized by a permanent cell cycle arrest that restricts the cell’s proliferative potential [29]. Many studies have shown a positive correlation between senescence in ADSCs and a reduction in the therapeutic effect of those cells.

Senescence determines reduced cell proliferation and viability, the significant deterioration of mitochondria, and inadequate defense against oxidative stress. Additionally, senescent cells release pro-inflammatory and matrix-degrading factors known as the senescence-associated secretory phenotype (SASP) [30].

Therefore, we will review the causes and biological mechanisms underneath the senescence of ASDCs. Furthermore, we will discuss the possible pharmacological strategies to revert cell senescence and the current evidence hitherto available in the literature.

## 2. Cellular Senescence Phenotypes and Molecular Mechanisms of Senescence

**Figure 1:**
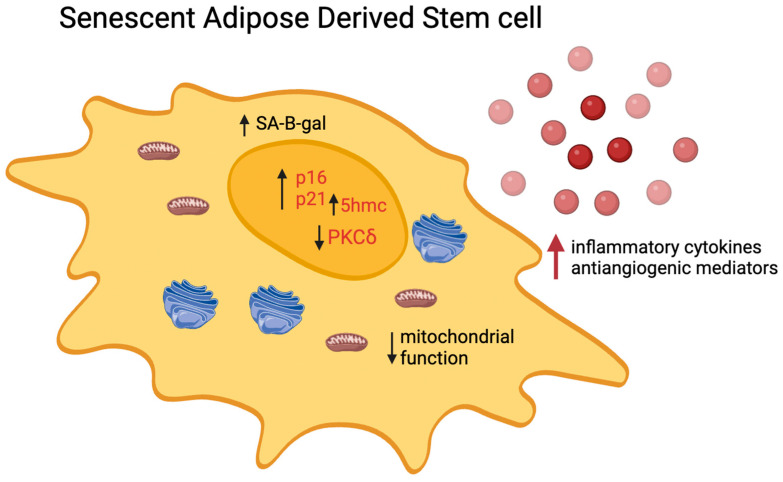
Pathways modulated in senescent ADSCs. Senescent ADSCs are identified by elevated levels of intracellular markers such as p16, p21, senescence-associated β-galactosidase (SA-β-gal), and 5-hydroxymethylcytosine (5hmc) while experiencing a decrease in PKC-δ levels. Additionally, these cells exhibit a senescence-associated secretory phenotype (SASP), which is characterized by an increased release of inflammatory cytokines (IL-1, IL-6, IL-8, MCP-1, and RANTES) and anti-angiogenetic mediators (IL-4, IFN-a2, IP10, IL-1b, PF4, DPPIV, and Activin A), further defining the altered functional state of senescent ADSCs.

A variety of internal and external stressors can induce cellular senescence. In response to these influences, cells activate a specific yet coordinated network of pathways that ultimately result in cell cycle arrest [31]. It is characterized by signaling, metabolic, and cytoskeletal alterations, leading to a decreased capacity of human adipose-derived stem cells (hADSCs) to handle stressors and DNA damage, and consequently affecting their therapeutic efficacy (Figure 1) [32]. Reportedly, these alterations culminate in a decline in tissue repair capabilities due to a sharp reduction in the self-renewal potential of hADSCs. Moreover, there is an augmented secretion of pro-inflammatory and matrix-degrading proteins and peptides, which carry local and systemic implications for overall tissue homeostasis. These context-dependent constraints could potentially limit the success of therapeutic regenerative outcomes [32,33]. Cellular senescence can be due, in vivo, to many pathological conditions and toxic stimuli. In vitro, it can be induced or accelerated by many factors which affect the cell division cycles.

In culture, primary cells have a finite capacity for division, typically undergoing approximately 40–60 cell cycles before reaching a state of proliferative cessation known as cellular senescence [34]. During this stage, cells remain viable but cannot proliferate further [31]. Cellular senescence is characterized by significant alterations in gene expression and the secretion of various cytokines, growth factors, and extracellular matrix modulators [33]. Apart from replicative exhaustion, the senescence of cultured stem cells can also be induced by other factors such as prolonged exposure to stressors like drug-induced DNA damage, radiation, hypoxia, or reactive oxygen species (ROS), as well as the presence of inflammation and inflammation-related chemokines and cytokines [35]. Therefore, it is crucial to consider the presence of senescent cells in cultures when conducting downstream cell-based assays or applying hADSCs in clinical settings, especially in older patients or patients affected by chronic diseases. This consideration is essential for maximizing treatment effectiveness and establishing therapeutic standards [36]. It is essential to identify senescent cells. Many authors have described markers that can be used to recognize and classify those cells.

Some of the markers commonly associated with cellular senescence are listed below [37].

### 2.1. Senescence-Associated β-galactosidase (SA-β-gal) Activity

The increased activity of SA-β-gal is a hallmark of senescent cells and is commonly detected using a histochemical assay [38]. The expression of pH-dependent senescence-associated β-galactosidase activity (SA-β-gal) exhibits a distinct difference between proliferating and senescent cells, making it a valuable indicator for identifying senescent cells [39]. Laboratory kits are commercially available to monitor SA-β-gal activity.

These kits typically provide all the necessary reagents and protocols for performing the SA-β-gal assay. The assay involves incubating cells with a substrate solution containing the β-galactosidase substrate, usually X-gal (5-bromo-4-chloro-3-indolyl-β-D-galactopyranoside), at an optimal pH (usually around pH 6) for the enzyme’s activity. Senescent cells, which have increased levels of SA-β-gal activity, will hydrolyze the substrate and produce a blue-colored precipitate, allowing for their visual identification under a microscope. Laboratory kits streamline the process of identifying this marker, ensuring consistency and reliability in detecting senescent cells based on SA-β-gal activity. This assay is widely used for studying cellular senescence in various research settings [35,37,40]. SA-β-gal activity is widely used as a marker for cellular senescence. However, its expression can also occur in normal physiological conditions, such as in non-senescent cells, which compromises its specificity as a senescence indicator. Therefore, the accurate identification of senescent cells necessitates the use of a combination of senescence markers.

In a study conducted by Katarzyna Siennicka et al., the highest levels of SA-β-gal were observed in ASCs derived from long-term cultures of old donors. Comparatively, ASCs from older donors exhibited higher SA-β-gal levels than those from younger donors. Additionally, higher levels were noted in long-term cultures versus short-term cultures. Statistical analysis indicates that the differences in SA-β-gal levels between young and old donors are less significant than the differences between short-term and long-term cultures, demonstrating that this phenomenon is independent of the donor’s age [41].

The increase in β-galactosidase activity in senescent cells reflects their elevated lysosomal content and is widely used as a marker for senescence. However, this method has certain disadvantages, such as a high rate of false positives in confluent or terminally differentiated cells. The test’s low specificity and variations in culture conditions, like cell confluence, can lead to an overestimation of the percentage of senescent cells [42]. EdU is a nucleotide analog that can be incorporated into MSC cultures for 24 h, marking cells undergoing DNA replication. Utilizing EdU staining helps mitigate some of the false positives associated with β-galactosidase staining. If a nucleus is positive for EdU labeling, indicating active DNA replication, the cell is not counted as positive for β-galactosidase staining, even if there are other markers typically associated with senescence [42]. Intense SA-β-GAL activity is also observed in cells with inherently high lysosomal β-GAL activity, such as macrophages, as well as in a variety of post-mitotic cells, including neurons, even during early embryonic development [43]. Positivity for β-galactosidase cannot be used as a marker in senescent cells with defective lysosomal β-galactosidase activity [44].

### 2.2. Markers of Persistent DNA Damage

These include proteins such as γH2AX (phosphorylated histone H2AX) and 53BP1 (p53 binding protein-1), which accumulate at sites of DNA damage and are indicative of DNA damage response activation.

### 2.3. p21WAF1/CIP

This cyclin-dependent kinase inhibitor (CDKI) plays a central role in mediating cell cycle arrest in senescent cells. Its upregulation is a characteristic feature of cellular senescence.

### 2.4. Absence of Detectable Cellular Proliferation Activity

Senescent cells typically exhibit a lack of proliferation, which can be assessed by measuring the incorporation of nucleotide analogs such as bromodeoxyuridine (BrdU) or tritiated thymidine ([3H]-dT) into DNA. Diminished levels of BrdU incorporation can be quantified through immunofluorescence staining [32].

These markers collectively provide a comprehensive assessment of cellular senescence and are widely used in research to identify and characterize senescent cells.

Over the years, considerable effort has been devoted to studying and delving into the differences in outcomes depending on the age of the ADSC donor. As for pre-clinical studies, the literature confronting the differences between young and geriatric donors is plentiful. These studies have been conducted in both animals and humans.

Ye et al. compared orbital adipose-derived stem cells (OADSCs) from young and old patients. While the numbers of viable nucleated cells isolated from orbital fat samples were comparable between the two groups, they observed a decline in progenitor cell frequencies (measured by the ability to form colonies) with age, as evidenced by the colony-forming unit-fibroblast (CFU-f) assay results. OADSCs from young donors exhibited a higher capacity to form colonies containing larger cell numbers. Additionally, the proliferation rate of young OADSCs was significantly higher compared to aged cells. Moreover, OASCs from older patients displayed increased cellular senescence features, as indicated by higher expression levels of p21 and p53 mRNA, along with a more significant number of cells staining positive for senescence-associated β-galactosidase (SA-β-gal). An age-related decrease in OADSC differentiation potential towards adipogenic, osteogenic, and chondrogenic lineages was also observed.

Moreover, the authors analyzed the mRNA levels of the p21 and p53 genes, along with the activity of SA-β-gal, as biomarkers to evaluate the senescence of OADSCs. The mRNA expression of both senescence-related genes was notably higher in older patients than younger ones (*p* < 0.05). Consistent with the real-time PCR findings, an increased accumulation of SA-β-gal was observed in cells isolated from older donors with prominent lower eyelids [45].

In a 2016 study by Lara B Zajic et al., adipose tissues from five young (<5 years) and six geriatric (>10 years) cats were harvested and cryopreserved for the subsequent isolation and culture of ADSCs. The abilities of the ADSCs in culture, lymphocyte proliferation suppression, and senescence were compared. It was noted that ADSCs from geriatric cats took significantly longer to reach P2 (median 11 days, range 9–22 days) than those from young, healthy cats (median 7 days, range 6–8 days). The obtained results suggested an impaired proliferation of geriatric feline ADSCs at P2. In contrast, their ability to suppress mitogen-stimulated lymphocyte proliferation in vitro remained comparable to the ADSCs from young cats [46].

J. Bagge et al. investigated the effect of donor age on the cellular proliferation of equine BM- and ADSCs. Specifically, bone marrow- and ADSCs, along with dermal fibroblasts (used as a biological control), were harvested from horses spanning five age groups (n = 4, N = 60): newborn (0 days), yearling (15–17 months), adult (5–8 years), middle-aged (12–18 years), and geriatric (≥22 years). The authors observed that the cellular proliferation of equine BM- and ADSCs declined significantly in the geriatric cohort compared to the younger age groups. The donor age equally affected the proliferation levels in both MSC types. Analysis of the mRNA levels revealed an upregulation in tumor suppressors, apoptotic genes, and growth factors in MSCs from older horses, alongside a downregulation of some pro-cycling genes, with minor differences between cell types. These findings underscore the decline in the cellular proliferation of equine BM- and ADSCs with an advanced donor age. Notably, both MSC types exhibited robust in vitro proliferation in the age groups below 18 years [47].

Many human studies have attempted to better define the variances among donor age groups. For instance, the findings of a study by L. Lei et al. analyzing a cohort comprising individuals under the age of 20 exhibited significantly higher levels of metabolic activity than for those over 61. Notably, cells from younger subjects demonstrated superior proliferation and adhesion capabilities compared to those obtained from older individuals. Furthermore, the replication rate of the cells exhibited a gradual decline with age, with the <20 age group consistently displaying higher rates than the >61 age group. A discernible contrast was observed between the two distinct age cohorts [48].

Moreover, Choudhery et al. conducted a study to evaluate senescence using two methods: measuring the mRNA expression of the p16 and p21 genes and detecting SA-β-gal expression. Initially, two groups of donors (aged <40 years and >50 years) were examined. PCR analysis was used to assess the mRNA levels of the p16 and p21 genes, which are senescence markers. The results revealed a significantly higher expression of both genes in the older group (>50 years) compared to the younger group (<40 years). SA-β-gal activity, a commonly used biomarker for identifying senescent cells, was also found to increase with the age of the ADSC donors. Choudhary et al. also examined the SOD activity in ADSCs. The results indicated significantly higher SOD activity in young MSCs compared to aged cells, suggesting the presence of more robust antioxidant defense mechanisms in young MSCs. Indeed, reactive oxygen species (ROS) are hypothesized to play a role in the aging process; hence, age-related fluctuations in superoxide dismutase (SOD) activity could significantly impact aging. SOD is an antioxidant enzyme that catalyzes the conversion of superoxide radical anions (O2−) into hydrogen peroxide, which is further metabolized to O_2_ and H_2_O by enzymes like glutathione peroxidase and catalase.

Finally, in the same study, the influence of donor age on the growth features and maximum population doublings of ADSCs was examined. Growth curves were constructed based on cell counts documented at each passage, demonstrating an exponential growth phase succeeded by reduced proliferation until the halting of cell division. Statistical analysis revealed a notable disparity (*p* < 0.05) in total population doublings, with the doubling times elongating in correlation with the donor age advancement [49].

Senescent hADSCs undergo a significant downregulation of genes involved in cell cycle progression, contributing to their cell cycle arrest. They can be identified by their enlarged size and the activation of DNA damage response (DDR) pathways.

As hADSCs approach senescence, critical mediators of the DDR, such as the phosphorylated form of the histone variant H2AX (γH2AX) and p53 binding protein-1 (53BP1), exhibit characteristic persistent DNA damage foci. These foci become increasingly abundant, transitioning from rare in self-renewing hADSCs to nearly 90% in hADSCs nearing senescence. Monitoring the presence of these foci can be achieved through staining for markers such as 53BP1 and γH2AX, which provides a reliable method for tracking the progression of senescence in hADSCs [35,37,50]. During senescence, the proportion of cells exhibiting the 53BP1 and γH2AX markers increases to approximately 90% of the total cell population.

Cells’ nuclear morphology and architecture can change with age, and these changes may include an increase in nuclear area. Furthermore, this increase in nuclear area can serve as a marker for cellular senescence [27].

A paper by Patel et al. studied PKCδ (protein kinase C delta). This kinase regulates mitogenic signals to modulate molecular events underlying senescence. Elevated PKCδ levels were observed in obese ADSCs. Using PRKCD siRNA to target PKCδI and PKCδVIII isoforms, Patel RS et al. found an increased proportion of senescent cells in both subcutaneous (sc-ADSCs) and omental (om-ADSCs) ADSCs from obese donors. In om-ADSCs from lean patients transfected with PRKCD siRNA, the senescent cell percentage doubled compared to untreated om-ADSCs, and in general, the total number of senescent cells increased in all donor types. These results suggest a potential role of PKCδ in regulating transcription factors that are crucial for maintaining the stem cell niche. PKCδ seems to be a key mediator in regulating cell cycle arrest, thus preserving the staminal component and preventing its uncontrolled expansion and proliferative senescence [51].

Senescent cells release pro-inflammatory and matrix-degrading factors known as the senescence-associated secretory phenotype (SASP). The SASP involves the release of various cytokines, chemokines, growth factors, proteases, and lipids by senescent cells. The composition of this secretome varies based on the senescence trigger. While the SASP has beneficial effects like aiding immune system recruitment to premalignant lesions and supporting tissue repair, the secretion of pro-inflammatory factors such as IL-6, IL-8, MCPs (monocyte chemoattractant proteins), and MIPs (macrophage inflammatory proteins) can promote harmful outcomes like proliferation, angiogenesis, and inflammation, both locally and systemically. Persistent DNA damage signaling is linked to the SASP [52]. Multiple constituents of the SASP can induce senescence in adjacent non-senescent cells, a phenomenon termed paracrine senescence [53].

The role of epigenetics in aging has gained attention in efforts at understanding the process of cell aging. Many studies have revealed genome-wide and locus-specific disparities via cytosine methylation analysis. For instance, in a study led by J. Borkowska et al., higher levels of 5-hydroxymethylcytosine (5hmC) were observed in adipose stem cells from older individuals compared to younger ones. However, no significant association was found between 5hmC levels and age-related changes in ten-eleven translocation methylcytosine dioxygenases (TETs) expression. Notably, 5hmC levels were correlated with the population doubling time. The study identified 58 differentially hydroxymethylated regions, with hypo-hydroxymethylated regions showing approximately two-fold enrichment in CCCTC-binding factor binding sites. The accumulation of 5hmC in aged cells may be attributed to inefficient active demethylation due to altered TET activity and reduced passive demethylation owing to slower proliferation [54].

Wu SH et al. conducted a comparative study on the characteristics of infant and adult adipose-derived mesenchymal stem cells (ADSCs) in terms of their proliferation, senescence, antioxidative capacity, and differentiation potential. The study utilized infant ADSCs collected from excised polydactyly fat tissue, which is considered surgical waste, and adult ADSCs obtained from thigh liposuction. The findings indicated that the infant ADSCs exhibited superior proliferation capabilities, reduced senescence, and greater differentiation potential compared to their adult counterparts [55].

In a study conducted by Ren et al., ADSCs from elderly donors demonstrated reduced proliferation and migration capacities and exhibited a senescent phenotype. Aging was associated with the upregulation of inflammatory markers and alterations in the immunomodulatory properties of ADSCs [56].

Rennert RC and Sorkin M et al. demonstrated that aged ASCs have reduced capacities to promote neovascularization and skin wound healing [57].

ADSCs isolated from elderly donors exhibit significant limitations in their tissue repair capabilities. These cells demonstrate reduced proliferative and migrative abilities, diminished multilineage differentiation potential, increased senescent characteristics, and a proinflammatory secretome [58].

DUXAP10 was found to be a positive regulator of ASC senescence and disability induced by aging. DUXAP10 was also revealed to impair the phenotype and function of ASCs [59].

## 3. Main Causes of Accelerated Senescence

**Figure 2:**
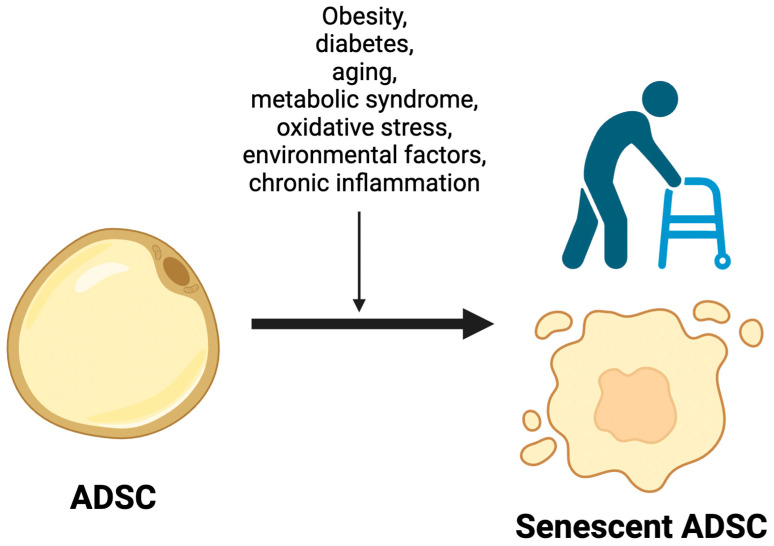
Factors promoting ADSCs’ senescence. Obesity, diabetes, aging, metabolic syndrome, oxidative stress, environmental factors, and chronic inflammation are considered key factors that promote increased senescence in adipose-derived stem cells (ADSCs). These conditions contribute to accelerated cellular aging and dysfunction, potentially impacting the regenerative capabilities of ADSCs.

Numerous causes of the senescence of adipose-derived stem cells are presently acknowledged in clinical practice and the scientific literature. Among these are obesity, diabetes, aging, metabolic syndrome, oxidative stress, environmental factors, and chronic inflammation, which have been extensively scrutinized over the years. (Figure 2)

For instance, Li et al. demonstrated that metabolic syndrome (MetS) influences the expression of senescence-associated (SA)-miRNAs in ADSc, as well as in ADSCs-derived extracellular vesicles (EVs), and subsequently affects the regulation of SA genes in pig models. They conducted an analysis of miRNAs in EVs derived from MetS-pig ADSc, revealing four upregulated miRNAs (miR-132, miR-199a-5p, miR-212, miR-374a-3p) and four downregulated miRNAs (miR-99b, miR-378, miR-504, miR-186) compared to EVs from lean-pig ADSc. These dysregulated miRNAs collectively target 5700 genes, 68 of which are associated with cellular senescence. Functional annotation clustering and pathway analysis of these 68 genes, which include MAPK1, PTEN, and MTOR, confirmed their involvement in cellular senescence, cell cycle regulation, metabolic processes, and MAPK signaling pathways. The authors also proved that metabolic syndrome diminishes the reparative ability of ADSCs-EVs both in vitro and in vivo [60].

ADSCs from donors with different BMI (Body Mass Index) values exhibit variations in their secretome, angiogenic potential, and migration capacity. Specifically, ADSCs from obese individuals show enhanced migration due to increased calpain-4, calpastatin, and MMP-15 expression.

Bunnell et al. demonstrated that ADSCs from obese individuals produce elevated levels of pro-inflammatory cytokines, contributing to the inflammatory milieu in obesity. Additionally, obesity alters the gene expression profile of ADSCs, with upregulation observed in genes such as leptin (LEP), leptin receptor (LEPR), sortilin 1 (SORT1), thyrotropin-releasing hormone (TRH), melanin-concentrating hormone 1 (MCHR1), peroxisome proliferator-activated receptor-gamma (PPAR-γ), peroxisome proliferator-activated receptor-gamma coactivator 1-α (PPARGC1A), and thyroid hormone receptor-β (THRB).

ADSCs from obese donors also produce higher levels of pro-tumorigenic factors, including leptin, IL-1, IL-6, IL-12, PDGF-A, TNF-*α*, leukemia inhibitory factor (LIF), intercellular adhesion molecule 1 (ICAM-1), and granulocyte-colony stimulating factor (GCSF), compared to ADSCs from lean individuals [61].

Adipose-derived stem cells (ADSCs) from lean individuals (L-ADSCs) and obese individuals (Ob-ADSCs) exhibit distinct biological properties. Specifically, Ob-ADSCs demonstrate reduced proliferation, differentiation, and immunomodulation abilities in vitro compared to L-ADSCs. This evidence suggests that obesity has an impact on the functional characteristics of ADSCs and could have implications for tissue repair and regeneration processes, as well as for overall health outcomes in obese individuals [62].

Obesity has been demonstrated to be related to mitochondrial and lysosomal dysfunction [63].

ADSCs from obese individuals (Ob-ADSCs) showed elevated levels of expression for CD36 and CD106 compared to those from lean individuals [64,65].

CD36 is a transmembrane glycoprotein known for facilitating the uptake of long-chain fatty acids. This process may contribute to the accumulation of lipids within adipose tissue, characteristic of obesity. On the other hand, CD106, also known as vascular cell adhesion molecule-1 (VCAM-1), is involved in immune responses and the recruitment of leukocytes to sites of inflammation due to interacting with integrin alpha-4/beta-1, suggesting an increased propensity for inflammatory responses within the adipose tissue of obese individuals.

Moreover, ADSCs from lean individuals (L-ADSCs) can inhibit the polarization of pro-inflammatory M1-type macrophages while promoting the polarization of anti-inflammatory M2-type macrophages. Conversely, Ob-ADSCs demonstrate a contrasting behavior by promoting the polarization of pro-inflammatory M1-type macrophages.

This dysregulated immune response, characterized by the prevalence of pro-inflammatory macrophages, could contribute significantly to the development and progression of obesity and associated metabolic disorders.

Many studies have shown mitochondrial abnormalities in adipose-ADSCs from obese individuals (Ob-ADSCs). Notably, alterations in the mitochondrial structure have been observed, including morphological changes such as a slim size, swollen areas, and a decrease in mitochondrial mass and mtDNA content. Furthermore, disturbances in the mitochondrial homeostasis are evident, characterized by a decreased mitochondrial membrane fluidity, an imbalance in fusion/fission dynamics, and increased mitochondrial fragmentation in Ob-ADSCs [66,67]. The impaired mitochondrial morphology and structure in Ob-ADSCs likely contribute to mitochondrial dysfunction, ultimately leading to cellular injury and reduced cellular activity. Significant reductions in glucose uptake, glycolysis, extracellular acidification rate (ECAR), and oxygen consumption rate (OCR), as well as decreased levels of glycolysisand tricarboxylic acid (TCA) cycle-related enzymes and metabolites were found in Ob-ADSC compared to L-ADSCs.

Additionally, impaired mitochondria in Ob-ADSCs contribute to the increased production of reactive oxygen species (ROS), further exacerbating mitochondrial dysfunction and triggering inflammation. Mitochondrial ROS have been shown to activate inflammatory pathways such as the NF-kB signaling pathway, while inflammation, in turn, can impair mitochondrial biogenesis and function.

Interestingly, treatment with Mito-Tempo (MT), a mitochondrial ROS scavenger, has been shown to restore mitochondrial function and inhibit inflammation in injured cells. These findings highlight the complex interplay between mitochondrial dysfunction, ROS production, and inflammation in Ob-ADSCs, highlighting potential targets for therapeutic intervention in obesity-related pathologies. A downregulation of the AMP-activated protein kinase (AMPK) pathway is shown in Ob-ADSCs compared to L-ADSCs. The AMPK pathway is known to activate peroxisome proliferator-activated receptor gamma coactivator 1-alpha (PGC-1α), a key regulator of mitochondrial biogenesis and function.

AMPK activation typically stimulates PGC-1α, which, in turn, promotes mitochondrial biogenesis and enhances mitochondrial function. Thus, the reduced activation of AMPK in Ob-ADSCs may lead to decreased levels of PGC-1α, impairing mitochondrial biogenesis and function.

DNA damage in the mitochondria-related loci seems to be a key step in the accelerated senescence induced by obesity and metabolic syndrome. In experimental models examining ADSCs derived from the subcutaneous fat of obese patients, Eirin et al. identified important epigenetic alterations in the profile of the 5-hydroxymethylcytosine (5hmC) of mitochondria-related genes [68]. The changes in the epigenetic profile determined a phenotypic alteration in the mitochondria, involving structural and functional changes. Compared to those derived from lean patients, the mitochondria of ob-ADSCs presented an increased production of superoxide, with mitochondrial matrix dysfunction determined by decreased levels of fatty acid metabolites, matrix density, and reduced membrane potential. Moreover, 5hmc profile alterations in ob-ADSCs were associated with increased senescence, similar to that observed in ADSCs derived from elderly patients [69]. Similarly, increased levels of 5hmc in mitochondrial antioxidant genes were described in ADSCs derived from the subcutaneous fat of patients with metabolic syndrome, as much as mitochondria structural alterations [70].

In addition to mitochondrial dysfunction, increased lysosomal membrane permeability in Ob-ADSCs has been proven [62]. Furthermore, some lysosomes within Ob-ADSCs contained accumulated undegraded materials reminiscent of pathogenic phenomena observed in lysosomal storage diseases and certain neurodegenerative disorders [71].

Moreover, the structural changes in and functional decline of lysosomes are hallmarks of cellular senescence, referred to as age-related lysosome damage [72]. It has been hypothesized that lysosomal dysfunction in Ob-ADSCs leads to accumulated cellular waste and damaged organelles, disrupting cellular component recycling and disturbing cellular homeostasis. In summary, the observed lysosomal dysfunctions in Ob-ADSCs, characterized by increased membrane permeability, the accumulation of undegraded materials, and decreased autophagy ability, likely contribute to cellular dysfunction and compromise cellular homeostasis.

The relationship between diabetes and senescence has also been under the spotlight. Indeed, Shune Xiao et al. conducted a study that suggests diabetes compromises hADSCs’ activities and cellular functions, including miR-1248 downregulation, impacting the effectiveness of stem cell therapy. Tissue hypoxia under diabetic conditions drives these changes, implicating a role of the HIF-1α pathway. In summary, miR-1248 modulates hADSCs’ proliferation and angiogenesis under glucolipotoxic diabetic conditions by regulating the miR-1248/CITED2/HIF-1α pathway. Shune Xiao et al. identified miRNA-1248, via miRNA microarray analysis, as a regulator of core molecular pathways in hADSCs’ activity and differentiation. Glucolipotoxic conditions downregulate miRNA-1248 expression, inhibiting cell proliferation and angiogenesis by activating CITED2 and suppressing HIF-1α. These data unveil a novel miR-1248/CITED2/HIF-1α pathway critical in the diabetes-induced impairment of hADSCs’ wound healing capacity, offering a potential therapeutic target. Cell proliferation assays revealed reduced rates in glycation end products treated with G-hADSCs and diabetic hADSCs (D-hADSCs) versus normal cells (N-hADSCs). G-hADSCs and D-hADSCs displayed diminished migration markers (CXCR4, MMP2, MMP9), elevated reactive oxygen species (ROS), and decreased angiogenesis-related gene expression (VEGFα, FGF2, Angpt1, TGFβ) compared to N-hADSCs. The MiR-1248 expression was significantly lower in G-hADSCs and D-hADSCs versus N-hADSCs. Its overexpression in hADSCs promoted in vivo skin wound healing, indicating its pivotal role in regulating hADSCs’ functions [73].

In addition to the inherent disease-related decline observed in these cells, as demonstrated in the abovementioned articles, iatrogenic factors can disrupt and hasten the progenitor component, accelerating its senescence. A notably pertinent instance is represented by radiotherapy.

Indeed, Papadopoulou et al. observed a decline in stem cell characteristics in irradiation-induced prematurely senescent ADSCs, as evidenced by reduced expression of the mesenchymal cell surface markers CD90, CD73, CD44, and CD105 compared to young cells. Additionally, senescent ADSCs displayed a catabolic phenotype, with the upregulation of MMP-1 and MMP-13, known features of cell senescence that are crucial for tissue homeostasis [74].

It has also been demonstrated that the method of cell culture can significantly influence the senescence of ADSCs. Different culture conditions, such as the nutrient availability, oxygen levels, and passage number, can affect the proliferation, differentiation, and aging processes of these cells.

The study by Kim et al. demonstrated that human mesenchymal stem cells (MSCs) cultured under atmospheric oxygen tension (21% O_2_) exhibit an increase in cell senescence markers compared to those cultured under low physiological in vivo oxygen tension [75]. Additionally, Ratushnyy et al. have demonstrated that human adipose-derived stem cells (ADSCs) exhibit elevated levels of senescence-associated β-galactosidase (SA-β-gal) activity and an increased expression of tumor suppressor genes p16, p21, p53, and pRb when cultured at atmospheric oxygen concentration (21% O_2_) compared to those cultured at 2–5% O_2_ [76].

Oxygen is essential for tissue cells due to its crucial role in energy metabolism. Considering that the oxygen concentration in stem cell niches in vivo is significantly lower than the 21% commonly used in cell culture systems, hypoxia treatment has been employed to modulate various functions of mesenchymal stem cells (MSCs). Zhihong Xu et al. demonstrated that continuous hypoxic culture not only significantly enhanced the proliferation capability but also mitigated the senescence of mesenchymal stem cells [77].

## 4. Senescence-Induced Functionality Changes in ADSCs

Factors like diabetes can alter the outcomes of ADSC applications [78]. Stem cell therapy for diabetic wounds is less effective compared to that for general wounds, likely due to glucolipotoxicity, oxidative stress, and hypoxia at the injury site, which decrease stem cell viability [79,80] Irregular glucose fluctuations contribute to reactive oxygen species (ROS) production, affecting the transplanted stem cells’ viability [81]. Additionally, ADSCs from diabetic hosts have an impaired biological function and reduced wound healing ability. Studies confirm that diabetic host-derived ADSCs have a lower healing capacity than those from non-diabetic hosts [82,83].

Moreover, in a recent study, G-hADSCs and D-hADSCs displayed lower proliferation rates and impaired wound healing abilities than N-hADSCs, as assessed by the CCK-8 and scratch wound assays. These findings suggest that glucolipotoxicity inhibits the proliferation, migration, and wound-healing capacities of G-hADSCs and D-hADSCs. Furthermore, G-hADSCs and D-hADSCs exhibited a diminished osteogenic differentiation potential but an enhanced adipogenic differentiation potential compared to N-hADSCs. Additionally, the angiogenesis-promoting effect of G-hADSCs and D-hADSCs was significantly reduced compared to N-hADSCs. Overall, these results indicate that glucolipotoxicity compromises the angiogenesis and multipotent differentiation potential of G-hADSCs and D-hADSCs when compared to N-hADSCs [73].

In the same study, on day 7, the skin wound diameter was significantly reduced in groups treated with N-hADSCs, G-hADSCs, or D-hADSCs. However, N-hADSC-treated rats showed lower wound diameters than those treated with G-hADSCs and D-hADSCs. By day 14, wounds in the G-hADSCs and D-hADSCs groups further decreased, whereas the N-hADSC-treated wounds tended to be nearly healed.

Histological examination on day 14 revealed enhanced tissue regeneration in rats treated with N-hADSCs, G-hADSCs, and D-hADSCs compared to PBS-treated rats. Hematoxylin and eosin staining showed inferior epithelialization and dermal rupture in the PBS group, complete epithelialization and a thicker dermis in the N-hADSCs group, and partial epithelialization with a thicker dermis in G-hADSCs and D-hADSCs groups.

Immunohistochemical staining at day 14 indicated reduced angiogenesis in the G-hADSCs and D-hADSCs groups compared to the N-hADSCs group. These results suggest that glucolipotoxicity diminishes the efficacy of G-hADSCs and D-hADSCs in treating skin wounds [73].

The donor age significantly influences the efficacy of ADSC therapies. In a 2013 study on multiple sclerosis, ADSCs from older donors (>60 years) failed to alleviate neurodegeneration in experimental autoimmune encephalomyelitis (EAE) mice, contrary to ADSCs from younger donors (<35 years). Mice treated with ADSCs from older donors showed increased CNS inflammation, demyelination, and splenocyte proliferation compared to those receiving cells from younger donors. These results underscore the critical role of donor age in determining ADSC-mediated neuroprotection, immunomodulation, and remyelination in EAE [84].

A study exploring age-related changes in the regenerative potential of ADSCs from fat pads in human lower eyelids found that ADSCs from protruded fat pads in older women exhibit diminished regenerative abilities due to reduced progenitor cell numbers and increased cellular senescence. This leads to prolonged proliferation times and decreased multilineage differentiation capabilities. The study suggests alternative strategies like cell banking or allogeneic cell sources for future ADSC-based therapies in elderly patients [45].

The alteration in ADSCs’ functionality based on senescence due to donor aging, or in vitro replicative senescence, is a topic of great interest and has been extensively studied in the literature.

Indeed, in another study, age significantly influenced the growth kinetics and population doubling time of the examined groups, revealing notable differences between young and elderly patients. Surprisingly, there were no significant changes in proliferation activity among older patient groups, suggesting that, while cell proliferation diminishes with age, hADSCs retain some proliferative potential even in older individuals. However, due to slower proliferation rates in cells from older patients, extended in vitro culture and expansion may be necessary before clinical application, potentially prolonging the timeline for autologous clinical use in these patients. Reduced proliferation rates and a reduced viability of cells may eventually lead to cell senescence and apoptosis. Although these findings indicate a decrease in the chondrogenic differentiation potential of hADSCs with age, this decline does not necessarily imply a loss of regenerative capacity. Across all groups, a robust proteoglycan-rich extracellular matrix and characteristic chondrocyte-like cell morphology were observed. However, significant differences were noted in the concentration of chondrogenesis markers at both the protein and mRNA levels. Given the rising prevalence of degenerative bone and joint diseases with age, the decline in chondrogenic and osteogenic differentiation potential associated with aging may pose limitations for the therapeutic utilization of hADSCs [85].

Moreover, a study by Ratushnyy et al. [86] suggested that the replicative senescence of ADSCs reduces the angiogenic potential of their secretome. This effect was observed using an in ovo blood vessel growth model, which incorporates various cellular and non-cellular components involved in angiogenesis. In this model, it was noted that the secretome from senescent ADSCs exhibited a diminished ability to support blood vessel growth compared to non-senescent ADSCs. Interestingly, no significant differences were observed between senescent and non-senescent ADSCs when evaluating other aspects of angiogenesis, such as the tubular structure formation in Matrigel and human umbilical vein endothelial cells’ (HUVECs) migration. These findings indicate that the impairment in angiogenic potential associated with replicative senescence may be specific to certain aspects of the angiogenic process or manifest differently depending on the experimental model used. Overall, these findings highlight the complex and context-dependent nature of the effects of replicative senescence on ADSCs’ function, particularly regarding angiogenesis. Replicative senescence results in increased paracrine activity in human ADSCs. Specifically, a significant increase in the production of inflammatory cytokines such as IL-6, IL-8, MCP-1, and RANTES was observed in senescent ADSCs. Moreover, many cytokines with proangiogenic activity were elevated in senescent ADSCs. Conversely, several secreted proteins with antiangiogenic activity, including IL-4, IFN-a2, IP10, IL-1b, PF4, DPPIV, and Activin A, were also increased. Additionally, the downregulation of specific growth factors and protease genes associated with the positive regulation of angiogenesis, such as IGF1, MMP1, TGFB3, PDGFRB, and PGF, was noted in senescent ASCs. Remarkably, no significant differences were observed in the VEGF concentration and expression between the early (passages 2–5) and late passages (passages 19–28) of senescent ADSCs. A decreased expression of genes whose products are involved in vascular formation (PGF, PDGFRB, and TGFB3) and a significantly increased expression of angiogenesis inhibitor genes (TIMP3, TIMP2, and ADAMST1) was found in senescent cells.

Similar data were demonstrated in a few studies of ADSCs isolated from the tissues of young and elderly donors [87,88].

The use of ADSCs to treat cardiovascular diseases and fight cardiac remodeling has been thoroughly explored. Candidate patients are often elderly, thus ADSC senescence could be a variable that should be considered for treatment success. A recent study compared ADSCs and BMSCs from young and elderly donors for use in surgical ventricular restoration. Elderly donor ADSCs showed higher proliferation potential and anti-aging abilities than BMSCs from the same age group. Immunohistochemical staining indicated the better survival of elderly ADSCs than BMSCs from the same donors. Moreover, immunofluorescence revealed increased vessel formation in patches seeded with elderly ADSCs, attributed to their robust proliferative and anti-aging properties. ADSCs may release specific cytokines which aid revascularization and cardiac remodeling. They also demonstrated superior anti-aging and angiogenic capacities compared to BMSCs in elderly donors. Notably, ADSCs showed enhanced biocompatibility with collagen patches, facilitating MSC localization on rat hearts post-implantation. These findings suggest that ADSC-seeded collagen patches with covalently immobilized bFGF and VEGF hold promise for surgical ventricular restoration (SVR) in elderly patients [89].

Another factor impacting ADSC applications is obtaining cells from obese donors. As mentioned above, obesity determines an accelerated cellular senescence, which may impair cell functionality. The literature extensively examines this aspect through numerous comparative studies aimed at understanding ADSC uses and distinctions. For example, a study demonstrates that MSCs derived from the adipose tissue of lean patients exhibit a superior capability to enhance cortical kidney perfusion compared to those from obese patients. Lean-MSCs also outperform obese-MSCs in reducing blood pressure, preserving kidney function, mitigating peritubular capillary loss, and remodeling vascular walls. Interestingly, lean and obese MSCs improve medullary kidney perfusion and oxygenation in RAS mice compared to vehicle-treated ones. Moreover, the reduced reparative capacity of obese MSCs in recipient mice kidneys is associated with obesity-induced senescence in donor MSCs, suggesting obesity’s role in impairing MSCs’ reparative function [90]. In addition, a few studies in mice have proven that weight loss can partially restore the cell proliferation, viability, and regenerative properties of ADSCs from formerly obese mice [91].

Regarding outcomes of ADSC-based therapies, like fat grafting, it is unclear how the burden of an increased number of senescent ADSCs could impact clinical results. Varghese et al. conducted a study to understand how various patient characteristics influence the outcomes of fat grafting procedures. Their research covered a spectrum of factors, including age, body mass index (BMI), gender, menopausal status, donor site location, and whether the patient had undergone cancer treatments such as radiotherapy, chemotherapy, or tamoxifen. The study aimed to determine how these factors affect the viability and functionality of adipocytes and adipose-derived stem cells (ADSCs), which are essential for the success of these procedures. The findings suggested that the yield of ADSCs remains fairly consistent across different age groups, and that BMI has a minimal impact on ADSC quality. However, the effects of gender, menopausal status, and hormone replacement therapy on ADSC yield have not been specifically studied yet. Additionally, no clinical studies have definitively identified the best donor sites for fat grafts. The research also highlighted that ADSCs are susceptible to the damaging effects of radiotherapy, indicating the need for careful consideration in clinical applications [36].

## 5. Senolytic Agents and Their Applications

ADSCs possess many notable properties, showcasing their potential in regenerative medicine and tissue repair. However, it is essential to acknowledge that they are still adult stem cells, which inherently have limitations in their self-renewal capabilities.

The absence or minimal presence of telomerase activity leads to a depletion of approximately 50–200 nucleotides following each instance of cell division [92].

Telomere shortening persists in vivo, yet its pace notably accelerates under conditions of proliferation. As cells undergo proliferation, their proliferative and differentiation capacities gradually diminish, leading to cellular senescence. This decline diminishes their suitability for medical applications. Senescent ADSCs halt cell division while maintaining metabolic activity. Nevertheless, these cells progressively enlarge due to functional and molecular alterations, adopting a “fried egg” morphology, exhibiting a reduced expression of specialized markers, and exhibiting diminished differentiation potential. Hence, it is imperative to discern the optimal procedural measures in ASC cultivation to optimize their clinical effectiveness [93].

Senescence, increasingly acknowledged as a pivotal mechanism in aging, involves cells under stress or experiencing damage entering a non-proliferation state. This transition leads to the buildup of senescent cells, marked by elevated levels of reactive oxygen species, senescence-associated β-galactosidase, senescence-associated heterochromatin foci, and a senescence-associated secretory phenotype (SASPs). These elements fuel inflammation, protein degradation, and tissue malfunction, perpetuating a cascade of senescence in neighboring cells and tissues. Furthermore, introducing aged or senescent cells has hindered the upkeep, rejuvenation, and restoration of musculoskeletal tissues in vivo. Consequently, the regenerative potential of mesenchymal stem cells (MSCs) faces limitations due to the challenge of establishing a clinically effective MSC population via in vitro isolation and expansion while concurrently addressing the detrimental accumulation of senescence [94].

Non-pharmacological approaches to inverting cellular senescence have been attempted and proved efficacious. Specifically, caloric restriction, physical exercise, and intermittent fasting increased mitochondrial function in the adipose tissue through the SIRT-1 and AMPK pathways. Furthermore, they decreased senescence-related markers like p16INK4 and NF-kB. However, these approaches need time to be effective in a clinical setting and are often hard to apply to a geriatric population [91].

The decline in the therapeutic efficacy of ADSCs due to aging and prolonged culture expansion has sparked interest in the potential use of senolytic agents to rejuvenate previously discarded cell donations.

This strategy involves attenuating senescence processes and fostering the enrichment of a more potent stem cell population. Furthermore, numerous naturally occurring senolytic agents, such as piperlongumine, dasatinib/quercetin, and fisetin, have demonstrated the ability to target and eliminate senescent cells by acting on anti-apoptotic pathways that are typically upregulated in these cells [95].

In a study conducted by Mullen et al., the potential of fisetin was investigated, particularly in mitigating the age-related degeneration of cartilage and bone in a progeria mouse model. Indeed, the researchers observed that fisetin functions in a dose-dependent manner, selectively diminishing these markers of senescence while conserving the differentiation capacity of the expanded ADSCs [94].

Yousefzadeh et al. (2018) found fisetin to have the best senolytic effect among the 10 flavonoids tested. Both in a specific cell subgroup of a progeroid mice model and omental human adipose tissue explants, fisetin downregulated senescence markers like IL-6, IL-8, and MCP-1. In older wild-type mice, fisetin helped restore tissue, extended mice’s lifespan, and decreased old age pathology [96].

In a study by Zhao et al., quercetin (Que) was studied as a senolytic agent. The researchers observed that it modulates tert-butyl hydroperoxide (TBHP)-induced changes in nucleus pulposus-derived mesenchymal stem cells (NPMSCs), such as diminished cell proliferation capacity, heightened SA-β-gal activity, cell cycle arrest, ROS accumulation, and the elevated expression of senescence-associated proteins. Notably, these indicators of senescence were significantly mitigated following Que treatment. Que reduced the expression levels of senescence-related proteins (p16, p21, and p53), as well as senescence-associated secretory phenotype (SASP) factors, including IL-1β, IL-6, and MMP-13, while concurrently increasing SIRT1 expression. Moreover, the protective effects of Que against cell senescence were partially reversed upon miR-34a-5p overexpression and SIRT1 knockdown. In vivo, X-ray and histological analyses assessed that Que ameliorated intervertebral disc degeneration (IDD) in a puncture-induced rat model [97].

Islam MT et al. found that dasatinib and quercetin (D&Q) exhibit senolytic properties by reducing age-related increases in markers of cellular senescence such as β-galactosidase, the expression of the p16 and p21 genes, and the P16 protein in perigonadal white adipose tissue. This treatment also reduced the expression of a pro-inflammatory SASP (senescence-associated secretory phenotype) gene group, including MCP1, TNF-α, IL-1α, IL-1β, IL-6, CXCL2, and CXCL10. Furthermore, it decreased crown-like structures and the prevalence of T cells and macrophages. However, in the liver and skeletal muscle, the effects of D&Q were not as pronounced. Overall, the study suggests that D&Q can reduce inflammation in adipose tissue and enhance the metabolic function in older individuals [98].

In an open-label Phase I pilot study, the combination of dasatinib and quercetin (D&Q) was investigated, and it has been observed that it selectively targets and eliminates senescent cells by transiently disrupting pro-survival networks. In the first clinical trial of senolytics, D&Q improved the physical function in patients with idiopathic pulmonary fibrosis (IPF), a fatal senescence-associated disease. In an open-label Phase I pilot study, oral dasatinib (100 mg) and quercetin (1000 mg) were administered to individuals with diabetic kidney disease for three days (N = 9; average age: 68.7 ± 3.1 years; 2 female; BMI: 33.9 ± 2.3 kg/m^2^; eGFR: 27.0 ± 2.1 mL/min/1.73 m^2^). Adipose tissue, skin biopsies, and blood were collected before and 11 days after completing senolytic treatment. The study found a reduction in the adipose tissue senescent cell burden within 11 days, with decreases in p16INK4A- and p21CIP1-expressing cells, cells with senescence-associated β-galactosidase activity, and adipocyte progenitors with limited replicative potential. Adipose tissue macrophages and crown-like structures were also decreased. The levels of skin epidermal p16INK4A+ and p21CIP1+ cells were reduced, along with circulating SASP factors, including IL-1α, IL-6, and MMPs-9 and -12. This “hit-and-run” treatment with senolytics significantly decreases the senescent cell burden in humans [99].

Glasstetter LM et al. interrogated the dysregulated 5hmC profile of senescent ADSCs derived from obese pigs and identified locus-specific changes in cell proliferation and fate pathways. Some of the 5hmC changes in genes corresponding to these pathways were reversed by vitamin C. Importantly, the proof-of-concept was extended from swine to human MSCs [69].

Therefore, research fields on this topic are ever open and continuously need fresh insights and novel scientific endeavors.

For instance, HMGA1 is pivotal in establishing senescence-associated heterochromatic foci, indicating its significant role in cellular senescence processes. Conversely, adipocyte differentiation is regulated by FOXO1 and SOD2, highlighting their importance in adipogenesis. Additionally, FOXO4 has been implicated in cell senescence by blocking p53-induced apoptosis through direct binding, underscoring its potential as a target for the development of senolytic medications [100].

Moreover, in a study on the angiogenic potential of ADSCs in patients with preeclampsia (PE), MSCs showed an increased expression of senescence-associated secretory phenotype (SASP) components, including interleukins-6 and -8 and MCP-1, following exposure to TNF-alpha in vitro. Subcutaneous adipose tissue staining revealed a heightened inflammatory response in preeclampsia, indicated by elevated levels of TNF-alpha and MCP-1 compared to normotensive pregnancies (*p* < 0.001 and 0.024, respectively). ADSCs from preeclampsia patients exhibited reduced cell viability and proliferation, and increased migration. Preeclampsia-derived ADSCs demonstrated a senescent phenotype at baseline, with increased staining for senescence-associated β-galactosidase (SABG), upregulated senescence markers and SASP components, and diminished angiogenic potential compared to normotensive pregnancy-derived MSCs. Dasatinib treatment significantly increased the apoptosis in preeclampsia-derived ADSCs compared to normotensive pregnancy-derived ADSCs and decreased the gene expression of p16 and six SASP components. The mechanistic link between senescence and impaired angiogenesis in preeclampsia was confirmed by the enhanced angiogenic potential of preeclampsia-derived ADSCs following dasatinib treatment [101].

The most recent studies about senolytic agents are listed in Table 1.

In conclusion, even though early results are promising, it is evident that there is still a significant need for research on senolytic agents, particularly within the continuously evolving and innovative realm of adipose-derived mesenchymal stem cells.

However, in some cases, ADSCs can revert senescence in specific cell types. A study by Lv et al. used ADSCs to perform anti-aging treatments on senescent cells and progeroid animal models. They found that mouse ADSCs, when cocultured with mouse embryonic fibroblast (MEF), had their senescence delayed and showed increased viability compared to MEF cultured alone with the same passage. The replicative senescence was delayed when MEF cocultured with ADSCs switched its metabolic homeostasis from catabolism to anabolism. Under the effect of ADSCs, MEFs were pushed to start mitophagy, removing damaged mitochondria and accumulated ROS. A similar result was obtained in vivo. PolG knocking mice (PolG mut/mut) were used as a model of progeria. PolG mut/mut mice had higher levels of brain and heart tissue mitophagy markers when treated with mADSC compared to the WT group and displayed improved alopecia and kyphosis. These data suggest that allogeneic stem cell therapy can improve aging-related symptoms via mitochondrial quality control [102].

## 6. Conclusions

ADSCs’ high availability and multilineage differentiation capacity have shown relevant therapeutic potential in regenerative medicine and tissue engineering. Because such promising applications often involve the treatment of chronic diseases and conditions found in an aging population, they might be hindered by stem cell senescence and dysfunction due to the natural aging process related to patient age or comorbidities. These features must be accounted for to enhance the efficacy of procedures involving ADSCs. The mechanisms of senescence are multiple and redundant, involving many cell structures, from the nuclear DNA to the mitochondria. Furthermore, conditions like diabetes, obesity, chronic inflammation, or metabolic syndrome could accelerate the aging process, which becomes independent of the biological age of the patient.

Our review suggests that ADSC populations with many senescent cells may present a reduced regenerative potential. Furthermore, elderly patients or patients with specific chronic diseases often present decreased progenitor cell numbers and increased cellular senescence, which may contribute to ADSCs’ prolonged proliferative time and reduced multilineage differentiation capabilities. However, the clinical significance of cell survival and the therapeutic efficacy of cell-based strategies in patients presenting a high number of senescent cells is still unclear. Many factors may contribute to the discrepant conclusions, such as the sample size, donor gender, age, and different experimental parameters and conditions; hitherto, few efforts have been made to eliminate these confounding variables.

Many strategies to revert the senescence process have been attempted. Senolytic drugs have often been used in vitro and on animal models with promising results, but their clinical translation is yet to be completed. Our review suggests that ASCs from elderly, diabetic, metabolic syndrome, and obese patients may be less desirable for regenerating such tissues. Alternative strategies such as cell banking, allogeneic cell sources, or cell-free products may be more appealing when developing future ADSC-based therapeutic approaches for patients at risk of increased cellular senescence. Nonetheless, the first step is fully understanding the clinical impact of senescence on cell-based approaches through large, tailored clinical studies involving at-risk patient populations.

## Figures and Tables

**Table 1:** Recent Literature on Senolytic Drugs.

Authors and Year of Publication	Type of Cells	Experimental Model	Key Findings	Experimental Settings	Reference
Yousefzadeh MJ, et al. 2018	Murine and human fibroblasts	Various flavonoid polyphenols were tested for their senolytic activity on senescent fibroblasts from mice and humans, which had been induced by oxidative and genotoxic stress.	Fisetin has been shown to possess senotherapeutic properties, demonstrating effectiveness in reducing senescence in both mouse models and human tissue samples.	In vitro	[96]
Mullen M, et al. 2023	hADSCs	Fisetin usage to Attenuates Cellular Senescence Accumulation During Culture Expansion	Fisetin has been effective in eliminating senescent cell populations from both early and late stage cell cultures without compromising their ability to differentiate into multiple cell types.	In vitro	[94]
Zhao WJ, et al. 2023	Rat-ADSCs	Usage of Quercetin in treating oxidative stress-induced senescence in rat cells	Quercetin ameliorates oxidative stress-induced senescence in rat nucleus pulposus-derived mesenchymal stem cells via the miR-34a-5p/SIRT1 axis	In vitro	[97]
Hickson LJ, et al. 2019	hADSCs	Usage of Dasatinib plus Quercetin	Dasatinib plus Quercetin usage decrease senescent cells in individuals with diabetic kidney disease	In vivo	[99]
Islam MT, et al. 2023	Mice ADSCs	Twenty-one-month-old mice were administered Dasatinib and Quercetin through oral gavage for three consecutive days every two weeks over a period of three months.	Dasatinib and quercetin exhibit senolytic properties, effectively reducing the age-related accumulation of senescence markers.	In vitro	[98]
Suvakov S, et al. 2019	hADSCs	hASCs exposure to TNF-alpha in vitro	Targeting senescence improves angiogenic potential of adipose-derived mesenchymal stem cells in patients with preeclampsia.	In vitro	[101]
Glasstetter LM, et al. 2023	Swine and Human ADSCs	5hmC profiles were examined through hydroxymethylated DNA immunoprecipitation sequencing followed by an integrative gene set enrichment analysis.	467 hyper- and 591 hypo-hydroxymethylated loci in swine Obese- versus Lean-ASCs were found and were partly reversed in swine Obese-MSCs treated with vitamin-C.	In vitro	[69]
